# Characterization of perineural invasion in basal cell carcinoma

**DOI:** 10.1016/j.jdin.2026.07.003

**Published:** 2026-07-18

**Authors:** Sa Rang Kim, Joseph Sarhan, Laura A. Valaas, Simon F. Roy, Stefano G. Daniele, Mariam B. Totonchy, Kathleen C. Suozzi

**Affiliations:** a Department of Dermatology, Yale University, New Haven, Connecticut; b Department of Dermatology, University of Washington, Seattle, Wasshington; c Department of Pathology, McGill University, Montréal, Canada; d Department of Dermatology, Massachusetts General Hospital, Harvard Medical School, Boston, Massachusets; e Skin Surgery Center, Seattle, Washington

**Keywords:** basal cell carcinoma, epidemiology, frozen sections, Mohs micrographic surgery, perineural invasion

*To the Editor:* Perineural invasion (PNI) is associated with high-risk basal cell carcinoma (BCC),^1^ yet data remain limited regarding the histologic patterns of PNI in BCC and the impact of PNI on clinical outcomes. To date, most studies on PNI in BCC report binary readouts of the presence of PNI.^2^^,^^3^ Here, we analyze the histopathologic features of BCC-associated PNI identified on frozen sections during Mohs micrographic surgery (MMS) that may better inform risk stratification.

Twenty-six cases of BCC with PNI were retrospectively identified at 2 clinical practices between 2019 and 2025 by querying Mohs operative reports for documented PNI, with cumulative incidence rate of 0.38%. Records were reviewed for demographics and tumor histology on initial biopsy. All frozen section slides obtained during MMS were reviewed for detailed PNI characterization and independently confirmed by a board-certified dermatopathologist. Nerve involvement was defined according to our group’s prior study on cutaneous squamous cell carcinoma with PNI.^4^ Evaluated features included nerve diameter, depth, number of involved nerves, intra- versus extra-tumoral nerve involvement (involvement within or separate from the bulk of the tumor), and circumferential nerve involvement (>50% encasement of the nerve sheath) (Fig 1). Two-sample *t*-tests were used for continuous variables and *P* value <.05 was considered statistically significant.

**Fig 1:**
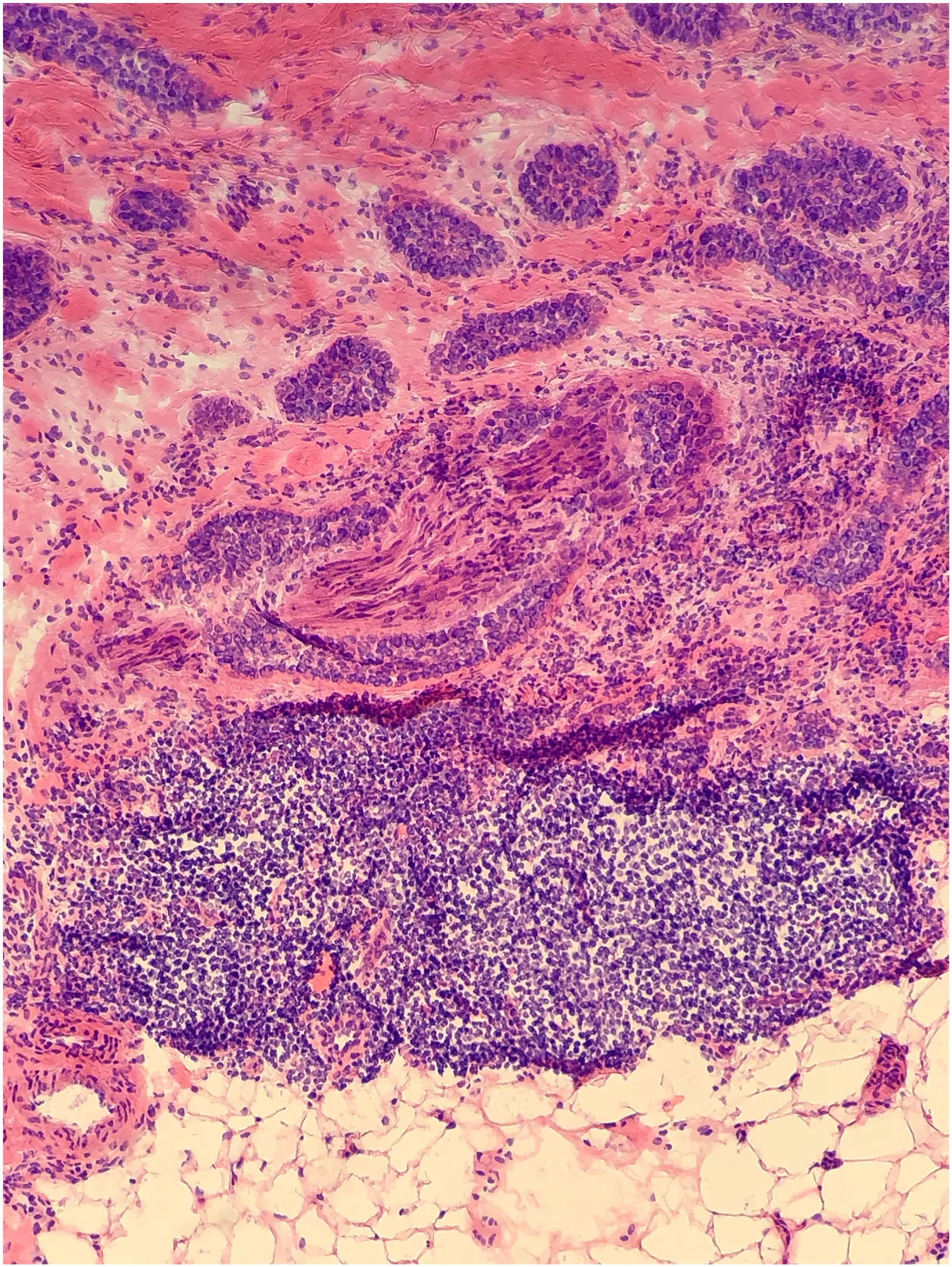
Micronodular basal cell carcinoma with perineural invasion (PNI). There is circumferential involvement.

Mean patient age was 66 years (Table I). Mean preoperative tumor size was 2.1 cm (range, 0.5-4.0), and mean postoperative defect size was 3.1 cm (range, 1.2-7.5). Four patients (15%) required additional excision under general anesthesia to achieve negative margins. All four cases demonstrated large-caliber PNI with extension into subcutis or muscle. Five cases (19%) initially classified as nodular or superficial histology on biopsy demonstrated infiltrative histology on Mohs sections. The mean largest nerve caliber was 0.11 mm (range 0.04-0.40; SD 0.08), and the mean number of involved nerves was 2.2 (range 1-6; SD 1.3). Depth of nerve invasion (dermis vs subcutis or deeper) was not associated with caliber or number of involved nerves. Six cases (23%) demonstrated extra-tumoral PNI, all with subcutaneous or deeper tumor extension. Tumors with infiltrative, micronodular or morpheaform histology on Mohs sections were associated with larger caliber nerves (0.11 vs 0.06 mm; *P* = .0042). Two patients received adjuvant radiation, and no adverse outcomes, defined as local recurrence or metastasis, were observed during available follow-up (mean, 176 days; range, 10-1070 days for 23 patients). Two recurrent tumors demonstrated no PNI on initial MMS but showed PNI at recurrence.

**Table I:** Summary of patient characteristics and histopathologic features of basal cell carcinoma with perineural invasion

Pt	Age (y)	Sex	Histology on MMS frozen section	Tumor location	Preoperative tumor size (cm)∗	Post-operative defect size (CM)∗	Deepest depth of nerve involvement	Largest nerve caliber (mm)	Number of nerves involved	Follow-up (d)
1	70	M	Infiltrative + micronodular	H/N	4.0	7.5	Subcutis	0.23	2	41
2	77	M	Infiltrative	H/N	1.5	2.6	Muscle	0.05	1	367
3	52	M	Infiltrative	H/N	4.0	4.6	Muscle	0.10	2	401
4	62	M	Infiltrative	H/N	2.1	3.1	Subcutis	0.13	2	58
5	52	F	Morpheaform+infiltrative	H/N	1.3	1.9	Subcutis	0.11	2	187
6	58	M	Nodular	H/N	2.0	2.9	Subcutis	0.05	2	37
7	32	M	Infiltrative	H/N	2.0	3.0	Dermis	0.15	6	94
8	86	F	Micronodular	H/N	1.4	2.0	Dermis	0.08	1	211
9	87	F	Nodular	H/N	2.4	3.0	Dermis	0.06	1	217
10	81	M	Nodular	H/N	4.0	4.3	Dermis	0.05	2	28
11	27	F	Infiltrative	H/N	0.9	1.4	Dermis	0.09	1	132
12†	41	F	Infiltrative	H/N	1.2	2.3	Dermis	0.10	1	93
13	75	M	Infiltrative + basosquamous	Trunk	3.0	4.2	Muscle	0.18	2	10
14†	67	M	Infiltrative	H/N	2.5	4.1	Dermis	0.09	2	1070
15	91	M	Infiltrative	Trunk	3.0	4.2	Dermis	0.10	1	32
16	91	F	Infiltrative	H/N	2.3	3.5	Subcutis	0.10	2	N/A
17	54	M	Infiltrative	H/N	1.1	3.0	Subcutis	0.06	2	158
18	52	F	Morpheaform	H/N	1.6	2.5	Dermis	0.04	1	N/A
19	83	M	Infiltrative	Extremity	0.8	1.7	Dermis	0.27	5	322
20	84	M	Nodular	H/N	2.0	2.5	Subcutis	0.10	2	32
21	67	M	Infiltrative	H/N	2.0	2.6	Subcutis	0.10	1	24
22	67	M	Infiltrative	Trunk	2.5	3.5	Dermis	0.10	3	N/A
23	63	F	Nodular	H/N	0.5	1.2	Dermis	0.06	4	336
24	72	M	Infiltrative + micronodular	H/N	3.0	4.3	Muscle	0.40	3	29
25	68	M	Infiltrative	H/N	1.4	3.1	Dermis	0.06	4	169
26	55	M	Infiltrative	H/N	1.0	2.0	Subcutis	0.06	2	10
MEAN	66				2.1	3.1		0.11	2.2	176

*H/N*, Head and neck; *MMS*, Mohs micrographic surgery.

∗ Largest diameter measured.

† Recurrent case.

In one case-matched study, PNI involving unnamed nerves was not an independent prognostic factor for BCC outcomes.^5^ In our cohort, large-caliber nerve involvement was common (58%) and associated with aggressive histologic subtypes and increased surgical complexity, including the need for additional excision under general anesthesia. Therefore, we believe PNI in BCC should be documented during MMS, as large-caliber PNI may identify more complex cases requiring multidisciplinary discussion. These findings should be interpreted with caution given the small sample size. Additionally, although no adverse outcomes were observed in our cohort, follow-up duration was limited and variable, and the prognostic implications of these findings remain uncertain.

### Declaration of generative AI and AI-assisted technologies in the writing process

Artificial intelligence tools were used solely for language refinement during manuscript preparation. All analyses, interpretations, and conclusions were performed by the authors, who take full responsibility for the manuscript content.

## Conflicts of interest

None disclosed.
